# Human herpesvirus 8 (HHV-8) detected by nested polymerase chain reaction (PCR) in HIV patients with or without Kaposi’s sarcoma. An analytic cross-sectional study: Herpesvírus humano 8 (HHV-8) detectado por reação da polimerase em cadeia do tipo *nested* PCR em pacientes HIV-positivos com ou sem sarcoma de Kaposi. Um estudo transversal analítico

**DOI:** 10.1590/1516-3180.2014.8973010

**Published:** 2015-04-14

**Authors:** Paula Renata Lima Machado, Kleber Juvenal Silva Farias, Maira Gabriela Martins Pereira, Patrícia Pereira da Silva de Freitas, Benedito Antônio Lopes da Fonseca

**Affiliations:** I PhD. Biomedical Scientist, Virology Research Center, Faculdade de Medicina de Ribeirão Preto (FMRP), Universidade de São Paulo (USP), Ribeirão Preto, São Paulo, Brazil.; II MSc. Biologist, Virology Research Center, Faculdade de Medicina de Ribeirão Preto (FMRP), Universidade de São Paulo (USP), Ribeirão Preto, São Paulo, Brazil.; III PhD. Veterinarian, Virology Research Center, Faculdade de Medicina de Ribeirão Preto (FMRP), Universidade de São Paulo (USP), Ribeirão Preto, São Paulo, Brazil.; IV MD, PhD. Associate Professor, Virology Research Center, Faculdade de Medicina de Ribeirão Preto (FMRP), Universidade de São Paulo (USP), Ribeirão Preto, São Paulo, Brazil.

**Keywords:** Sarcoma, Kaposi, Herpesvirus 8, human, HIV, Polymerase chain reaction, Serology

## Abstract

**CONTEXT AND OBJECTIVE:**

Kaposi’s sarcoma (KS) is a common neoplastic disease in AIDS patients. The aim of this study was to evaluate the frequency of human herpesvirus 8 (HHV-8) infection in human immunodeficiency virus (HIV)-infected patients, with or without KS manifestations and correlate HHV-8 detection with KS staging.

**DESIGN AND SETTING:**

Analytic cross-sectional study conducted in a public tertiary-level university hospital in Ribeirão Preto, São Paulo, Brazil.

**METHODS:**

Antibodies against HHV-8 lytic-phase antigens were detected by means of the immunofluorescence assay. HHV-8 DNA was detected in the patient samples through a nested polymerase chain reaction (nested PCR) that amplified a region of open reading frame (ORF)-26 of HHV-8.

**RESULTS:**

Anti-HHV-8 antibodies were detected in 30% of non-KS patients and 100% of patients with KS. Furthermore, the HHV-8 DNA detection rates observed in HIV-positive patients with KS were 42.8% in serum, 95.4% in blood samples and 100% in skin biopsies; and in patients without KS, the detection rate was 4% in serum. Out of the 16 serum samples from patients with KS-AIDS who were classified as stage II, two were positive (12.5%); and out of the 33 samples from patients in stage IV, 19 (57.6%) were positive.

**CONCLUSION:**

We observed an association between HHV-8 detection and disease staging, which was higher in the serum of patients in stage IV. This suggests that detection of HHV-8 DNA in serum could be very useful for clinical assessment of patients with KS and for monitoring disease progression.

## RESUMO

**CONTEXTO E OBJETIVO:**

Sarcoma de Kaposi (SK) é uma doença neoplásica comum em pacientes com aids. O objetivo deste estudo foi avaliar a frequência da infecção por herpesvírus humano 8 (HHV-8) em pacientes infectados por HIV, com ou sem SK e associar a detecção do HHV-8 com o estadiamento do SK.

**TIPO DE ESTUDO E LOCAL:**

Estudo transversal analítico realizado em hospital universitário público terciário de Ribeirão Preto, São Paulo, Brasil.

**MÉTODOS:**

Anticorpos contra antígenos de fase lítica do HHV-8 foram detectados por imunofluorescência. O DNA viral de HHV-8 foi detectado em amostras de pacientes pela reação em cadeia da polimerase do tipo *nested* (*nested* PCR), que amplificou uma região do fragmento de leitura aberta (ORF)-26 do HHV-8.

**RESULTADOS:**

Anticorpos anti-HHV-8 foram detectados em 30% dos pacientes sem SK e 100% dos com SK. Além disso, a detecção de HHV-8 DNA observada em pacientes HIV-positivos com SK foi de 42,8% no soro, 95,4% em amostras de sangue e 100% em biópsias de pele, e em pacientes sem SK foi de 4% no soro. Das 16 amostras de soro de pacientes com SK-AIDS classificados como estádio II, duas foram positivas (12,5%) e, das 33 amostras de pacientes no estádio IV, 19 (57,6%) foram positivas.

**CONCLUSÃO:**

Observamos associação entre a detecção do HHV-8 e o estadiamento da doença, que foi maior no soro de pacientes no estágio IV. Isso sugere que a detecção do HHV-8 no soro poderia ser muito útil para a avaliação clínica de pacientes com SK e para o monitoramento da progressão da doença.

## INTRODUCTION

Human herpesvirus 8 (HHV-8) is associated with three neoplastic disorders: Kaposi’s sarcoma (KS), primary effusion lymphoma (PEL) and multicentric Castleman’s disease.^1^^,^^2^^,^^3^ KS is an angioproliferative disease that is particularly frequent and aggressive in patients with AIDS. It commonly presents as multifocal disease, frequently in the upper body, head and neck, with a rapid course regarding both local progression of lesions to tumors and visceral dissemination, leading to organ dysfunction and high mortality.^4^ The most common sites for visceral involvement by KS are the lungs (37%), gastrointestinal tract (50%) and lymph nodes (50%).^5^

Since HHV-8 has not been isolated in cell cultures, HHV-8 infection is identified by means of either serological methods or molecular biology assays. Several qualitative and quantitative amplification techniques for HHV-8 detection in different biological samples have been developed.^6^^,^^7^^,^^8^ HHV-8 viral sequences have been successfully detected by means of the polymerase chain reaction (PCR) in various specimens, such as in KS lesions. HHV-8 sequences can also be detected in plasma and in peripheral blood mononuclear cells with very high specificity and sensitivity. The HHV-8 viral load in peripheral blood mononuclear cells of KS patients has been shown to correlate with tumor burden, but this approach has only in frequently been used to monitor KS patients in clinical practice.^9^

Although high rates of HHV-8 antibodies (19.6-57.4%) have been found in Brazilian Amerindians,^10^^,^^11^ only low rates of HHV-8 antibodies have been found in blood donors from other parts of Brazil (2.8-7.4%).^12^^,^^13^ The HHV-8 antibody prevalence among healthy children and young adults in different cities in the state of São Paulo ranges from 1.0 to 4.1% in different age groups. Among AIDS patients, the prevalence has been found to be 39.2% (51/130).^14^

## OBJECTIVES

The aims of the present study were to evaluate the frequency of HHV-8 infection in human immunodeficiency virus (HIV)-infected patients, with or without KS manifestations, and to assess the association between HHV-8 detection and KS staging.

## METHODS

This analytic, cross-sectional study was conducted in the Infectious Disease Unit of the University Hospital of Ribeirão Preto, São Paulo, Brazil. The subjects examined in this study comprised HIV-1 positive patients with KS, and HIV-1 positive patients without KS. The number of HIV patients was calculated as 50 (considering 5% alpha, test power of 80% and prevalence of HHV-8 infection of at least 5%). The sample size was calculated using the PS Power and Sample Size Calculations 2.1.30 software. The study was approved by the Ethics Committee of this institution (no. 12999).

The diagnosis of KS was clinically suspected and histologically confirmed. The histopathological criteria for diagnosing KS included spindle cell proliferation, erythrocyte-filled vascular slits and proliferation of small vessels, with vessels showing evidence of extracellular hemorrhage and hemosiderin deposition. KS staging was performed in accordance with the Mitsuyasu and Groopman system.^15^

BCBL-1 cells were cultured in RPMI medium with 20 ng/ml of 12-O-tetradecanoylphorbol-13-acetate (TPA; Sigma) for 96 hours. The cells were then washed twice with phosphate-buffered saline (PBS), placed on slides (10 µl/well), fixed in cold acetone for 10 minutes and stored at -20 °C. The slides were incubated with human serum (1:40 dilution in PBS with 3% fetal bovine serum) at 37 °C for 30 minutes. They were then washed, incubated with fluorescein isothiocyanate-conjugated goat anti-human immunoglobulin G (IgG) (Dako) at 1:256 dilution in Evans blue at 37 °C for 30 minutes, washed again and air dried. Coverslips were mounted with buffered glycerol. Whole-cell fluorescence in about 20% of the TPA-treated cells was considered positive for antibodies against lytic-phase antigens.

Nested PCR was performed on DNA extracted from serum, peripheral blood cells and skin tissue. DNA was extracted from 200 µl of serum and peripheral blood cells using the QIAamp DNA blood kit (QIAGEN) and from 25 mg of skin tissue using the QIAamp DNA tissue kit (QIAGEN), in accordance with the manufacturer’s instructions, and DNA was eluted with 50 µl of adding the elution buffer AE. For all PCR reactions, 5 µl of deoxyribonucleic acid (DNA) obtained from extraction of serum, peripheral blood cells or tissue were used.

In a total volume of 50 µl, the PCR mixture contained 0.2 mM of each dNTP, 25 pmol of each sense and antisense primer (5’-AGCCGAAAGGATTCCACCAT-3’ and 5’-TCCGTGTTGTCTACGTCCAG-3’)^1^, 1.5 mM of MgCl_2_, 2.5 U of Taq DNA polymerase (Invitrogen) and 5 µl DNA extracted from each sample. PCR amplification of HHV-8 was done at 94 °C for 1 minute, followed by 35 cycles of 1 minute at 94 °C, 1 minute at 58 °C and 1 minute at 72 °C, with a final extension step (10 minutes at 72 °C) to allow complete extension of the amplicons. The nested PCR amplification mixture contained 1 µl of the first PCR mixture and the same PCR reagents described above, except that 25 pmol of each sense and anti-sense internal primers (5’-TTCCACCATTGTGCTCGAAT-3’ and 5’-TACGTCCAGACGATATGTGC-3’)^1^ were used. Again, the amplification was done at 94 °C for 1 minute, followed by 40 cycles of 1 minute at 94 °C, 1 minute at 60 °C, 1 minute at 72 °C and a final extension of 10 minute at 72 °C. Ten microliters of each reaction mixture were analyzed by means of electrophoresis on 2% agarose gel. Positive reactions yielded an amplicon of 211 bp, which was easily viewed through ethidium bromide staining.

As a positive control, b-globin was amplified using PCR from each specimen (blood samples and skin tissue) using the primers GL1 and GR2.^16^ A set of negative controls (sterile water and a negative clinical sample) was included during all steps of the DNA isolation and amplification.

Recombinant plasmids containing amplicons of the HHV-8 ORF-26 were prepared in order to determine the nested-PCR sensitivity. The amplicon of 233 bp was amplified from a KS patient, purified using the Wizard SV Gel and PCR Clean-Up System kits (Promega), ligated to the pDrive cloning vector (QIAGEN) and used to transform *Escherichia coli* DH5α competent cells. The recombinant plasmids were then recovered using the Perfectprep Plasmid mini-kit (Eppendorf). Plasmids were sequenced using the ABI Prism Big Dye Terminator Cycle sequencing-ready kit (Applied Biosystems) with M13 forward or reverse primers, and the nucleotide sequence contained in this plasmid was compared with HHV-8 sequences retrieved from the GenBank database. These recombinant plasmids were used to analyze the absolute quantity of HHV-8 DNA detected by the nested PCR. Serial tenfold dilutions of a mixture containing a known copy of HHV-8 plasmids were amplified using nested PCR, and the analytical sensitivity of this technique was considered to be the last dilution presenting an amplicon band.

The variations of positivity for HHV-8 were determined using Fisher’s exact test, with a significance level of 0.05. Calculations were performed using the BioEstat 5.0 software.^17^

## RESULTS

Out of the 49 HIV-positive patients with a clinical or histopathological diagnosis of KS, 43 (87.8%) were males and 6 (12.2%) females, with ages ranging from 20 to 79 years (mean: 40 years). Out of the 50 HIV-positive patients without manifestations of KS who agreed to participate in this study, 36 (72%) were males and 14 (28%) females, ranging in age from 29 to 47 years (mean: 37.8 years).

Regarding the topography of AIDS-KS lesions, 32.7% of the patients presented cutaneous lesions alone; 30.6% cutaneous and digestive tract lesions; 16.3% cutaneous and respiratory tract lesions; 10.2% cutaneous, digestive tract and respiratory lesions; and 10.2% disseminated disease with lymph node involvement.

Out of the 49 patients, 16 were classified as stage II (more than one anatomical area and more than 10 cutaneous lesions) and 33 as stage IV (skin and visceral involvement). None of them were classified as stage I (one anatomical area with less than 10 lesions) or stage III (visceral involvement alone).

All of the AIDS-KS patients were positive for HHV-8 antibodies in the immunofluorescence assay (IFA), whereas only 15/50 of the patients without KS were positive (100% versus 30%).

The analytical sensitivity of nested PCR, analyzed in terms of the ability to detect plasmids containing the HHV-8 DNA sequence, was 17 copies of HHV-8 DNA. Forty-nine serum samples, 21 peripheral blood samples and 13 skin biopsies from patients with AIDS-KS, and 50 serum samples from HIV-positive/KS-negative patients were subjected to molecular detection of HHV-8. The HHV-8 DNA detection rates for HIV-positive patients with KS were 42.8% (21/49) in serum, 95.4% (21/22) in peripheral blood samples and 100% (13/13) in skin biopsies. In HIV-positive patients without KS, the detection rate for HHV-8 DNA was 4% (2/50) in serum **(**Table 1**)**. The efficiency of DNA extraction from blood samples and biopsies was confirmed by means of PCR amplification of the human β-globin gene.

**Table 1.**
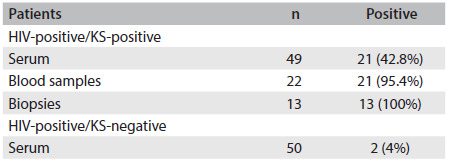
Prevalence of HHV-8 DNA in different samples from HIV-positive patients with and without Kaposi’s sarcoma (KS) as evaluated by nested polymerase chain reaction (nested PCR) test

HHV-8 = human herpesvirus 8; DNA = deoxyribonucleic acid; HIV = human immunodeficiency virus.

When broken down according to disease stage, out of the 16 serum samples from patients with AIDS-KS classified as stage II, two (12.5%) were positive for HHV-8 through PCR. Out of the 33 samples from patients in stage IV, 19 (57.6%) were positive (P = 0.0048) **(**Table 2**)**. Thus, it was seen that HHV-8 DNA was more readily detected in patients with disseminated disease.

**Table 2.**
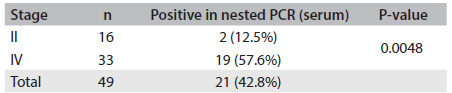
Prevalence of HHV-8 DNA in serum samples from HIV-positive patients with Kaposi’s sarcoma according to stage as evaluated by nested polymerase chain reaction (nested PCR) test

HHV-8 = human herpesvirus 8; DNA = deoxyribonucleic acid; HIV = human immunodeficiency virus.

In the group of HIV-positive/KS-positive patients, it was possible to obtain 22 peripheral blood samples (19 from stage IV and 3 from stage II patients). Presence of HHV-8 DNA was detected in all stage IV patients and in two of the stage II patients. Analysis on these 22 peripheral blood samples according to their corresponding serum samples showed that nested PCR was positive in 12/19 and 1/3 of the stage IV and II patient samples (63.1% versus 33.3%), respectively. No significant difference was detected between these groups (P = 0.1364).

## DISCUSSION

Epidemiological studies have determined that HHV-8 seropositivity in various populations is strongly correlated with the population’s risk of developing KS^4^^,^^14^^,^^18^^,^^19^ and several longitudinal studies have shown that HHV-8 infection precedes the onset of KS.^4^^,^^13^^,^^20^ Among men who are infected with both HIV and HHV-8, the hazard ratio for KS is estimated to be 5.04%, and the 10-year probability of developing KS is up to 49.6%. The incidence of AIDS-KS has declined considerably since the use of highly active antiretroviral therapy (HAART) became widespread. The effect of the treatment may result from direct action of antiretroviral drugs on HIV, which is known to trigger KS, or may constitute direct antiviral action against HHV-8. Furthermore, immune reconstitution following HAART may lead to better recognition and clearance of HHV-8 through specific immune responses.^4^

Our study found HHV-8 antibody percentages of 30% and 100% in HIV-positive/KS-negative and HIV-positive/KS-positive patients, respectively. Other studies conducted in Brazil have found rates ranging from 13.9% to 39.2% in KS-free HIV-infected patients,^11^^,^^14^^,^^18^^,^^19^^,^^21^^,^^22^ from 80.0 to 98.7% in HIV-positive/KS-positive patients^14^^,^^19^^,^^21^ and from 2.5 to 25.1% in the general population.^14^^,^^19^^,^^23^^,^^24^^,^^25^ Several groups have reported variable seroprevalence rates for HHV-8 in the general populations of several countries. Depending on the assay used and the countries examined, the seroprevalence of HHV-8 antibodies ranges from 0 to 53% in the general population.^26^ These results should take into consideration the fact that the current serological tests present some accuracy problems with regard to detecting antibodies against HHV-8, especially in patients with asymptomatic infection.^27^ It should be pointed out that these results also vary with the type of immunofluorescence assay used, since the lytic IFA is more sensitive than IFA-latency-associated nuclear antigen (LANA) even though IFA-LANA is more specific than lytic IFA.^28^

HHV-8 DNA can be amplified from KS tissue at different clinical stages of the disease. Furthermore, semi-quantitative analysis has established that the HHV-8 DNA load is higher in patients with multicentric and visceral involvement than in those with localized disease, and also that the nodular stage is associated with a higher viral load than the patch and plaque stages, thereby showing a correlation between viral load and disease severity.^29^

There are conflicting reports regarding the prevalence of HHV-8 DNA in human tissues and body fluids, because most reports are based on data obtained through PCR of varying sensitivity and specificity. Nested PCR has been used to detect HHV-8 in paraffin-embedded tissues from biopsies on KS and multicentric Castleman’s disease cases, lymphoid tissues from PEL patients, semen, plasma, peripheral blood and saliva.^30^

In the present study, HHV-8 DNA was detected in 95.4% (21/22) of blood samples, thus indicating that HHV-8 can be found in peripheral blood mononuclear cells (PBMC) from individuals carrying HHV-8. In areas of low prevalence, such as the United Kingdom, France, United States and Canada, nested PCR has shown that there is no positive test in PBMC among healthy individuals. HHV-8 has been detected in PBMC in 9% of HIV-1 patients in parts of Italy. In Uganda, a country with high incidence of KS, HHV-8 was detected in PBMC in 14% of the total population.^30^ High frequency (82%) of detectable HHV-8 DNA in PBMC from 36 AIDS-KS cases from the Central African Republic was detected using a real-time PCR quantitative assay.^31^ The high prevalence of HHV-8 DNA found in the presence study might represent selection bias, since all of our patients presented very severe disease.

In a study on the molecular epidemiology of HHV-8 among Cuban and German patients with KS and asymptomatic sexual contacts, HHV-8 DNA was isolated from PBMC and amplified by means of nested PCR for ORF-K1 in 41.7% (10/24) of the cases of asymptomatic sexual contact.^32^

Out of the 49 serum samples from patients with AIDS-KS, 21 (42.8%) were HHV-8 positive in our study, thus indicating that these patients presented viremia, an important event in the pathogenesis of KS. Viral DNA was detected in 12.5% (2/16) of the serum samples from patients in KS stage II and 57.6% (19/33) of the patients in stage IV, thereby demonstrating that detection of HHV-8 in serum appears to be associated with disease stage. In HIV-positive patients without AIDS-KS, 4% (2/50) were positive in nested PCR, in agreement with the seropositivity obtained by means of HHV-8 IFA on the serum samples from these two patients. These patients certainly present higher risk of developing AIDS-KS.

Thus, nested PCR applied to serum samples can be used to assess the effects of HAART on HHV-8 viremia and of the specific drugs used for treating KS. In addition, detection of herpes virus DNA in lymphocytes could possibly represent latent infection, while detectable DNA in serum or plasma is usually associated with disease.^33^

The nested PCR applied in the present study was very sensitive: it was able to detect approximately 17 copies of HHV-8 and provided a sensitive and quick diagnosis. Thus, this test can be used for confirmation of histopathological examinations, especially in cases of early vascular lesions, in which histopathological diagnosis is difficult.

Detection of HHV-8 by means of PCR is important for making the differential diagnosis of KS and for therapy. Virus detection in serum may be very useful for clinical assessment of patients with KS and for monitoring disease progression, which is important for clinical practice, thereby avoiding unnecessary treatments. Finally, this study provides the basis for developing further studies on Kaposi’s sarcoma and HHV-8 in our hospital.

## CONCLUSION

The percentage of anti-HHV-8 antibodies, detected by lytic IFA, was 30% for non-KS patients and 100% for patients with KS, and we observed an association between HHV-8 detection and disease staging, which was higher in serum from patients in stage IV.
